# Prognostic value of computed tomography characteristics for overall survival in patients with maxillary cancer

**DOI:** 10.1186/s12885-016-2830-z

**Published:** 2016-10-10

**Authors:** Ying Yuan, Jingbo Wang, Yingwei Wu, Guojun Li, Xiaofeng Tao

**Affiliations:** 1 Department of Radiology, Shanghai Ninth People’s Hospital, Shanghai JiaoTong University School of Medicine, Shanghai, 200011 China; 2 Department of Head and Neck Surgery, The University of Texas MD Anderson Cancer Center, Houston, TX 77030 USA

**Keywords:** Computed tomography, Overall survival, Maxilla, Cancer

## Abstract

**Background:**

Our aim was to identify the preoperative computed tomographic (CT) characteristics most efficient in predicting overall survival (OS) of patients with maxillary cancer (MC).

**Methods:**

A retrospective review of CT images was performed in 115 patients with histopathologically confirmed primary MC from January 2005 to December 2013, who were classified into 2 subtypes (epithelial and non-epithelial) according to tissue of origin. The prognostic value of CT characteristics for OS was determined firstly through univariate Kaplan-Meier survival estimates with log-rank tests. Significant predictors were further tested with multivariable Cox proportional hazard models.

**Results:**

CT characteristics predictive of OS in univariate survival analysis were long and short diameter of the mass, long and short diameter of the largest cervical lymph node and adjacent soft tissue infiltration (*P* < 0.05). In the multivariable Cox analyses, the significantly independent predictors were long diameter of mass ≥ 4.2 cm (hazard ratio [HR] 1.8; 95 % confidence interval [CI] 1.1–3.0) and short diameter of the largest lymph node ≥ 7 mm (HR 1.9; 95 % CI 1.0–3.6) for all MC patients, as well as for non-epithelial MC patients (HR 3.1; 95 % CI 1.2–8.0; HR 3.3; 95 % CI 1.3–8.7, respectively).

**Conclusions:**

Preoperative CT characteristics of tumor size, lymph node size and adjacent structure infiltration are predictive of the OS time of MC patients. The information brought up in this study could be used in clinical practice to inform about the possible prognosis, and be beneficial to clinical decision making.

## Background

According to the annual report on status of cancer collected by the National Central Cancer Registry (NCCR) of China, approximately 39,450 new cases of oral cavity cancer were diagnosed in 2011, with 16,933 deaths occurring annually [1]. Estimated 5-year survival for primary oral cavity cancer was 71 % between 2003 and 2009, varying from 32.2 to 90.2 % depending on cancer location [2]. To date, no nationwide overall survival (OS) data for maxillary cancer (MC) has been reported in China and other countries. Cancers located in the maxilla may originate from odontogenic structures or jawbone, constituting from a broad histopathological spectrum of lesions, either epithelial or non-epithelial [3, 4]. Diversity in tissue of origin and exceedingly low prevalence bring difficulties in differential diagnosis and prognostic prediction.

Currently, computed tomography (CT) is the primary cross-sectional imaging tool clinically used to direct diagnosis, guide therapy and monitor treatment response of jaw lesions. Preoperative imaging would be used to inform about the possible prognosis, and is beneficial to clinical decision making. So far, the predictive value of CT variables for patient survival has been confirmed in invasive bladder cancer [5], lung cancer [6], hepatocellular carcinoma [7], and esophageal cancer patients [8]. Nevertheless, no relative studies have been conducted concerning utility of CT characteristics in predicting prognosis of patients with MC. Therefore, in the current study, we reviewed the patients from a retrospective database at our institution to evaluate overall survival time of MC patients and to investigate the association of preoperative CT characteristics with overall survival.

## Methods

### Patient selection

Our study retrospectively collected patients with pathologically proved MC, who underwent preoperative CT scan and received treatment in our institution from January 2005 to December 2013. Patients were excluded if they (1) received treatment (surgery or chemoradiation) for the cancer before CT scan; (2) had a previously diagnosed head and neck cancer; or (3) CT images could not be obtained or interpreted. The medical records of patients were reviewed and the following information was retrieved for analyses: age, gender, smoking status, alcohol use, histopathological results, TNM staging, and treatments. Patients were defined as “ever smokers,” if they smoked at least 100 cigarettes in their lifetime, and as “never smokers” otherwise. “Ever drinkers” were defined as those who drunk at least one alcoholic beverage per week for at least one year, and as “never drinkers” otherwise [9]. We further classified the patients into 2 subtypes according to the tissue of origin: epithelial and non-epithelial, by referring to the pathological classification published by the World Health Organization in 2005 [10]. The institutional review board of Shanghai Ninth People’s Hospital approved this retrospective study.

### CT Acquisition and analyses

In this study a 64-row helical CT system (Philips Brilliance, Philips Medical Systems, Best, the Netherlands) was used. Prior to treatment, the patients underwent CT examination within 1 week. The scanning parameters were 120–140 kV, 200–300 mA, 23 cm field of view, 256 × 256 matrix, and 5 mm section thickness. The patients were injected with iopamidol (Iopamiro 320, Bracco, Milan, Italy) or iopromide (Ultravist 300, Schering, Germany) at a dose of 1.5 mL/kg body weight by a power injector at a rate of 2.5 mL/s.

CT images were evaluated with Centricity Radiology RA 600 (version 6.1, GE Healthcare, Milwaukee, WI, USA) by three radiologists (Y.Y., Y.W. and X.T.) with more than 5 years of experience in head and neck radiology. All reviewers were blinded to histopathologic results. For continuous variables, the average of three radiologists’ measurements was adopted, including tumor size (long diameter of the mass [LM] and short diameter of the mass [SM]), lymph node size (long diameter of the largest cervical lymph node [LLN] and short diameter of the largest cervical lymph node [SLN]), CT value (CT value on plain image [plCT], CT value on contrast enhanced image (ceCT), and increase of CT value [inCT = ceCT - plCT]; by drawing 15–20 mm^2^ circular region of interest [ROI] on the most prominently enhanced portion of the mass). Each continuous variable was converted to binary variables with cutoff value of median for statistical analyses. Qualitative CT characteristics were also included and evaluated by consensus, including margin (well-defined [more than two-thirds of the margin was sharply demarcated]/ill-defined [less than one-third of the margin was sharply defined] [11]), cortical involvement (with/without maxillary cortical destruction) and soft tissue infiltration (with/without adjacent soft tissue infiltration [muscle, fat, or neurovascular structures]).

### Statistical analysis

The OS time was calculated from the preoperative CT examination date until death from any cause or the last follow-up date (Oct. 1, 2015). The prognostic value of CT characteristics for OS was determined through univariate Kaplan-Meier survival estimates with log-rank tests. Significant predictors were then tested with multivariable Cox proportional hazard models, and stratified analyses according to tissue origin. The estimated hazard ratio (HR) and 95 % confidence interval (CI) was adjusted for potential confounding effects, such as age, gender, smoking status, alcohol use, stage and treatments. Statistical analyses were carried out with STATA version 10.0 (College Station, TX). *P* < 0.05 was considered as statistically significant.

## Results

### Patients and clinical characteristics

A total of 115 patients (46 male, 69 female; mean age 50.0 ± 18.5 years) with histopathologically confirmed MC were reviewed, including 67 patients with epithelial MC (58.3 %) and 48 patients with non-epithelial MC (41.7 %). Pathologic diagnoses were as follows: squamous cell carcinoma (*n* = 26), osteosarcomas (*n* = 16), adenoid cystic carcinoma (*n* = 15), myofibroblastic sarcoma (*n* = 10), mucoepidermoid carcinoma (*n* = 7), ameloblastic carcinoma (*n* = 5), chondrosarcoma (*n* = 5), ghost cell odontogenic carcinoma (*n* = 3), malignant mixed tumor (*n* = 3), myoepithelial carcinoma (*n* = 3), spindle cell carcinoma (*n* = 3), undifferentiated high grade pleomorphic sarcoma (*n* = 3), adenocarcinoma (*n* = 2), Ewing’s sarcoma (*n* = 2), lymphoma (*n* = 2), malignant melanoma (*n* = 2), malignant peripheral nerve sheath tumor (*n* = 2), plasmacytoma (*n* = 2), giant cell carcinoma (*n* = 1), malignant fibrous histiocytoma (*n* = 1), malignant solitary fibrous tumors (*n* = 1) and rhabdomyosarcoma (*n* = 1). The clinical characteristics of patients are summarized in Table 1.

**Table 1:** Demographics and preoperative CT characteristics of MC patients (*n* = 115)

Characteristics	*n* (%)	Log-rank (*P* value*)
Gender		0.7472
male	46 (40.0)	
female	69 (60.0)	
Age (year)		**0.0114**
< 50	55 (47.8)	
≥ 50	60 (52.2)	
Smoking		0.7057
Ever	26 (22.6)	
Never	89 (77.4)	
Alcohol		0.7333
Ever	13 (11.3)	
Never	102 (88.7)	
Stage		0.4887
I-II	49 (42.6)	
III-IV	66 (57.4)	
T stage		0.8583
Low (T_0-1_)	45 (39.1)	
High (T_2-4_)	70 (60.9)	
N stage		0.7059
Low (N_0-1_)	79 (68.7)	
High (N_2-3_)	36 (31.3)	
M stage		0.0379
M_0_	100 (87.0)	
M_1_	15 (13.0)	
Treatment		**0.0010**
S	25 (21.7)	
S&C/X	81 (70.4)	
Other	9 (7.8)	
LM (cm)		**0.0072**
< 4.2	56 (48.7)	
≥ 4.2	59 (51.3)	
SM (cm)		**0.0058**
< 3.0	60 (52.2)	
≥ 3.0	55 (47.8)	
LLN (mm)		**0.0411**
< 12	56 (48.7)	
≥ 12	59 (51.3)	
SLN (mm)		<**0.0001**
< 7	55 (47.8)	
≥ 7	60 (52.2)	
plCT (HU)		0.4641
< 40	56 (48.7)	
≥ 40	59 (51.3)	
ceCT (HU)		0.0883
< 62	57 (49.6)	
≥ 62	58 (50.4)	
inCT (HU)		0.2441
< 20	58 (50.4)	
≥ 20	57 (49.6)	
Soft tissue infiltration		**0.0001**
Yes	86 (74.8)	
No	29 (25.2)	

*C* chemotherapy, *CI* confidence interval, *CT* computed tomography, *HR* hazard ratio, *HU* Hounsfield unit, *LLN* long diameter of the largest cervical lymph node, *LM* long diameter of the mass, *MC* maxillary cancers, *S* surgery, *SLN* short diameter of the largest cervical lymph node, *SM* short diameter of the mass, *X* radiotherapy

*P values of log-rank test for all MC patients

Bold number means statistically significant

### Effect of tissue of origin on OS

**Fig. 1:**
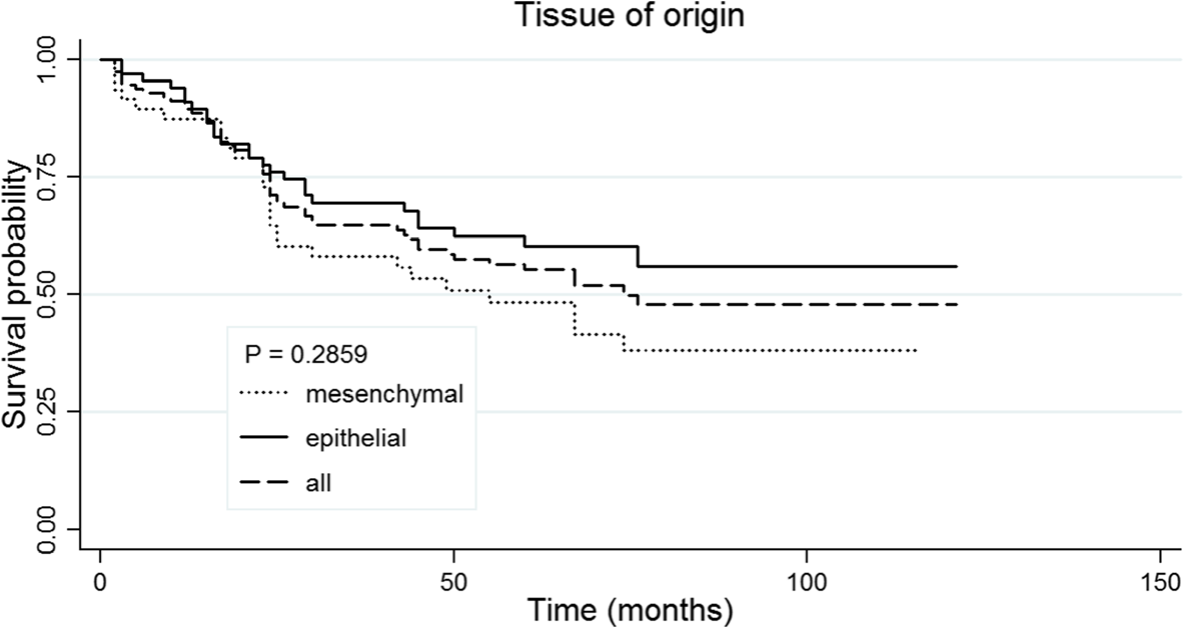
Kaplan-Meier curve of overall survival for MC patients according to tissue of origin

A total of 53 patients died during follow-up. The median follow-up time was 50 months (range: 2–121 months). The OS of all patients were 89.6 % (95 % CI: 82.4–93.9 %) at 1 year, 64.8 % (55.2–72.8 %) at 3 years and 55.4 % (45.4–64.2 %) at 5 years. The OS of epithelial MC patients at 1, 3 and 5 years were 91.0 % (81.2–95.9 %), 69.5 % (56.8–79.2 %) and 60.4 % (47.0–71.4 %); while the OS for non-epithelial MC patients were 87.5 % (74.3–94.2 %), 58.3 % (43.1–70.7 %) and 48.4 % (33.4–62.0 %), respectively. The Kaplan-Meier curves of OS for all MC patients, epithelial MC patients and non-epithelial MC patients are presented in Fig. 1. The OS rate of epithelial MC patients was higher than that of non-epithelial MC; however, no statistical difference was found (*P* > 0.05).

### Association of TNM staging and CT Characteristics with OS

**Fig. 2:**
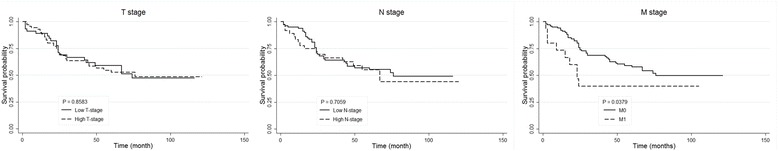
Kaplan-Meier curves of overall survival for T stage, N stage, and M stage. Low T stage: T_0-1_; high T stage: T_2-4_; low N stage: N_0-1_; high N stage: N_2-3_

**Fig. 3:**
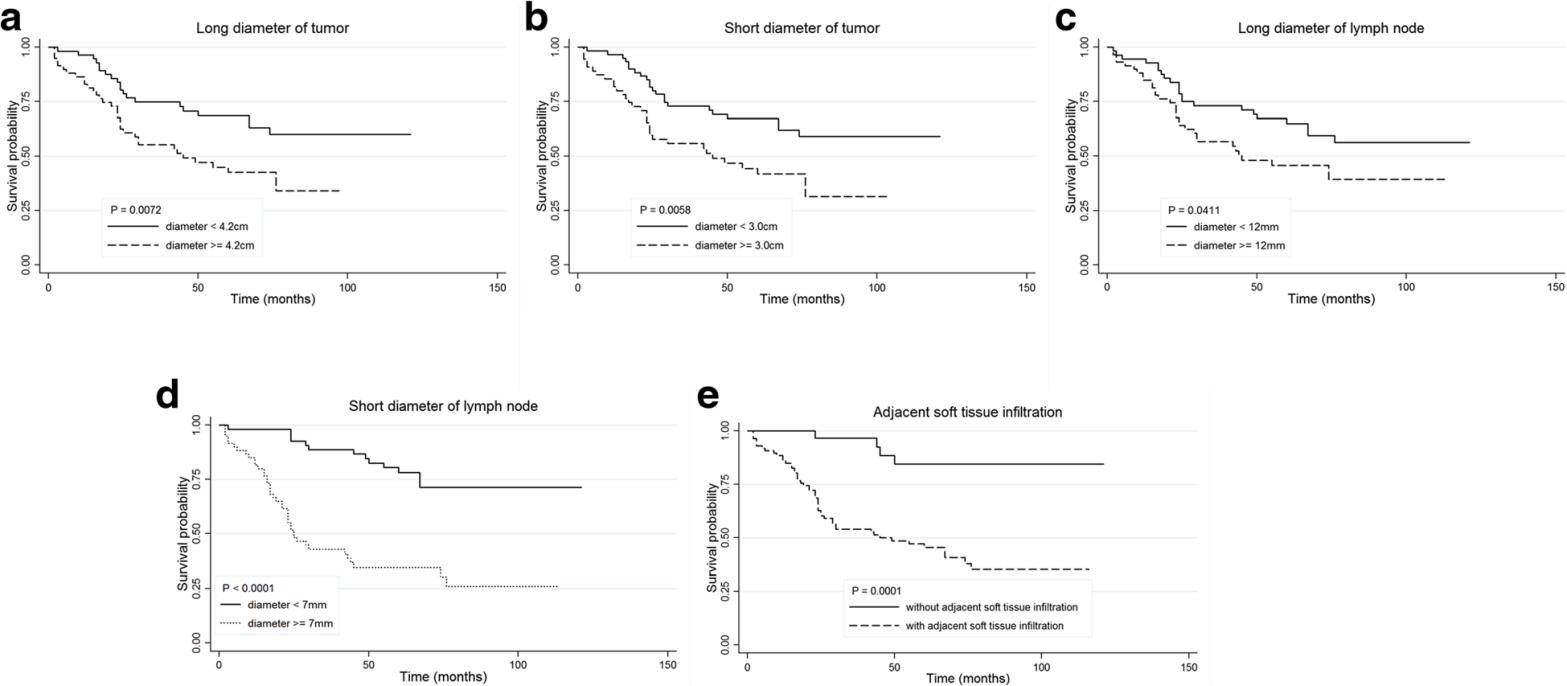
Kaplan-Meier curves of overall survival for CT characteristics: **a** long diameter of the tumor, **b** short diameter of the tumor, **c** long diameter of the largest cervical lymph node, **d** short diameter of the largest cervical lymph node, and **e** adjacent soft tissue infiltration

We retrospectively collected the TNM staging data according to clinical records of these patients. Kaplan-Meier survival curves for T stage (high versus low), N stage (high versus low) and M stage (M_0_ versus M_1_) were respectively evaluated. As shown in Fig. 2, we did not find significant effects of T and N stage on OS except for M stage. A statistically worse OS was experienced by M_1_ stage patients (*P* = 0.0379), while no statistical difference was found between patients with high and low T or N stages (*P* > 0.05). For CT characteristics, each continuous variable was converted into binary variables with medians as cutoff value (variable: LM, cutoff: 4.2 cm; SM, 3.0 cm; LLN, 12 mm; SLN, 7 mm; plCT, 40 Hounsfield unit [HU]; ceCT, 62HU; inCT, 20HU). In univariate log-rank analyses, a statistically worse OS was experienced by the patients with masses presenting adjacent soft tissue infiltration (*P* = 0.0001), LM ≥ 4.2 cm (*P* = 0.0072), SM ≥ 3.0 cm (*P* = 0.0058), LLN ≥ 12 mm (*P* = 0.0411), and SLN ≥ 7 mm (*P* < 0.0001), respectively. A total of 115 (100 %) and 112 (97.4 %) MCs demonstrated ill-defined margin and cortical destruction; therefore, no survival analyses were conducted on these two variables. The plCT, ceCT and inCT showed no significant predictive value (*P* > 0.05). The univariate log-rank results of CT characteristics for OS are summarized in Table 1. Kaplan-Meier curves of the significant CT predictors for OS are shown in Fig. 3a-e.

For multivariable Cox proportional hazard models, we first determined the main effects of significant predictors acquired from univariate log-rank analyses (continuous variables [LM, DM, LLN and SLN]; qualitative characteristics [adjacent soft tissue infiltration]) in all MC patients, and then stratified the data according to the tissue origin. As shown in Table 2, LM (HR 1.8; 95 % CI 1.1–3.0) and SLN (HR 1.9; 95 % CI 1.0–3.6) remained significant predictors in all MC patients, as well as in non-epithelial cancers (HR 3.1; 95 % CI 1.2–8.0; HR 3.3; 95 % CI 1.3–8.7, respectively). For patients with epithelial MC, none of the five CT characteristics were found predictive to overall death. Specifically for epithelial MC, our multivariable Cox proportional hazard models showed that the treatment, N stage and M stage were associated with OS (Table 3). Furthermore, the patients with SLN ≥ 7 mm were more likely to have higher T stages (OR, 2.3; 95 % CI, 1.0–4.8) (Table 4). Approximately 69.5 %, 33.9 % and 15.3 % of patients with LM ≥ 4.2 cm were diagnosed with high T stage, high N stage and M_1_ stage, respectively; while 70.0 %, 31.7 % and 16.7 % of patients with SLN ≥ 7 mm having high T stage, high N stage and M_1_ stage, respectively.

**Table 2:** Multivariable analyses of CT characteristics for OS

Characteristics	All MC patients (*n* = 115)			Epithelial MC (*n* = 67)			Non-epithelial MC (*n* = 48)		
	*n* (%)	*P* value	HR^a^ (95 % CI)	*n* (%)	*P* value	HR^a^ (95 % CI)	*n* (%)	*P* value	HR^a^ (95 % CI)
LM (cm)		0.022	**1.8 (1.1-3.0)**		0.392	1.4 (0.7-2.9)		0.017	**3.1 (1.2-8.0)**
< 4.2	56 (48.7)			34 (50.7)			22 (45.8)		
≥ 4.2	59 (51.3)			33 (49.3)			26 (54.2)		
SM (cm)		0.334	1.26 (0.8-2.0)		0.623	1.2 (0.6-2.7)		0.432	1.4 (0.6-3.1)
< 3.0	60 (52.2)			35 (52.2)			25 (52.1)		
≥ 3.0	55 (47.8)			32 (47.8)			23 (47.9)		
LLN (mm)		0.450	1.2 (0.7-2.0)		0.087	1.9 (0.9-3.8)		0.514	0.8 (0.4 ~ 1.6)
< 12	56 (48.7)			30 (44.8)			26 (54.2)		
≥ 12	59 (51.3)			37 (55.2)			22 (45.8)		
SLN (mm)		0.047	**1.9 (1.0-3.6)**		0.693	1.2 (0.6-2.5)		0.014	**3.3 (1.3-8.7)**
< 7	55 (47.8)			28 (41.8)			27 (56.3)		
≥ 7	60 (52.2)			39 (58.2)			21 (43.8)		
Soft tissue infiltration		0.984	1.0 (0.5-2.1)		0.994	1.0 (0.6-1.7)		0.862	1.1 (0.4-2.9)
Yes	86 (74.8)			51 (76.1)			35 (72.9)		
No	29 (25.2)			16 (23.9)			13 (27.1)		

*CI* confidence interval, *CT* computed tomography, *HR* hazard ratio, *LLN* long diameter of the largest cervical lymph node, *LM* long diameter of the mass, *MC* maxillary cancers, *SLN* short diameter of the largest cervical lymph node, *SM* short diameter of the mass

^a^Adjusted for potential confounding effect, such as age, gender, smoking status, alcohol use, stage and treatments

Bold number means statistically significant

**Table 3:** Multivariable analyses of clinial and CT characteristics for OS in epithelial MC patients (*n* = 67)

Variable	*P* value	HR (95 % CI)
age	0.858	0.9 (0.5-1.9)
gender	0.437	0.7 (0.4-1.6)
smoking	0.929	1.0 (0.4-3.0)
alcohol	0.806	0.9 (0.3-2.7)
treatment	**0.049**	**1.8 (1.0-3.3)**
T stage	0.618	0.7 (0.2-2.5)
N stage	**0.023**	**2.8 (1.2-6.7)**
M stage	**0.028**	**2.9 (1.1-7.7)**
soft tissue infiltration	0.458	1.4 (0.6-3.2)
LM	0.933	1.0 (0.5-2.4)
SM	0.157	1.9 (0.8-4.6)
LLN	0.234	1.6 (0.8-3.2)
SLN	0.779	1.1 (0.5-2.5)

*CI* confidence interval, *CT* computed tomography, *HR* hazard ratio, *LLN* long diameter of the largest cervical lymph node, *LM* long diameter of the mass, *MC* maxillary cancers, *SLN* short diameter of the largest cervical lymph node, *SM* short diameter of the mass

Bold number means statistically significant

**Table 4:** Association between TNM stage and CT Characteristics of LM and SLN in MC patients

CT characteristics	T stage			N stage			M stage		
	Low T(45) *n* (%)	High T(70) *n* (%)	OR (95 % CI)	Low N(79) *n* (%)	High N(36) *n* (%)	OR (95 % CI)	M_0_(100) *n* (%)	M_1_(15) *n* (%)	OR (95 % CI)
LM									
≥ 4.2 cm	18 (40.0)	41 (58.6)	2.1(.99- 4.5)	39 (49.4)	20 (55.6)	1.3 (0.6-2.8)	50 (50.0)	9 (60.0)	1.5 (0.5-4.5)
< 4.2 cm	27 (60.0)	29 (41.4)		40 (50.6)	16 (44.4)		50 (50.0)	6 (40.0)	
SLN									
≥ 7 mm	18 (40.0)	42 (60.0)	**2.3 (1.0-4.8)**	41 (51.9)	19 (52.8)	1.0 (0.5-2.3)	50 (50.0)	10(66.7)	2.0 (0.6-6.3)
< 7 mm	27 (60.0)	28 (40.0)		38 (48.1)	17 (47.2)		50 (50.0)	5 (33.3)	

*CI* confidence interval, *CT* computed tomography, *OR* odds ratio, *LLN* long diameter of the largest cervical lymph node, *LM* long diameter of the mass, *MC* maxillary cancers

Low T stage: T_0-1_; high T stage: T_2-4_; low N stage: N_0-1_; high N stage: N_2-3_

Bold number means statistically significant

## Discussion

The MCs may share clinical characteristics but have different prognoses [12]. CT is the primary imaging modality for preoperative evaluation of MC; however no report is available on the predictive value of CT findings on MC patients’ survival. Therefore, we attempted to find predictive factors for OS in MC patients using both quantitative and qualitative CT characteristics. The continuous variables, such as diameters of the mass (LM and SM), diameters of the largest cervical lymph node (LLN and SLN) and CT value (plCT, ceCT and inCT), are included because they are easily measured parameters and more reliable than others such as the imaging diagnosis of lymph node metastasis. Qualitative CT variables, such as margin, cortical involvement and adjacent soft tissue infiltration, are also clinically acceptable and easy to assess. Since almost all patients demonstrated ill-defined margin (100 %) and cortical destruction (97.4 %), no survival analyses were conducted with these two variables.

In the current study, univariate log-rank analysis showed that LM and SM were associated with OS of MC patients. A statistically worse OS was experienced by the patients with preoperative LM ≥ 4.2 cm and SM ≥ 3.0 cm. The multivariate Cox analysis confirmed that LM was the independent prognostic factor in all MC patients, particularly in non-epithelial MC. The predictive value of tumor size has been previously discussed in lung adenocarcinoma using cutoff values of 20, 30, 50 and 70 mm with a mean tumor size of 28.9 mm [6], in solitary small hepatocellular carcinoma with a mean tumor size of 26–27 mm [7], and in locally advanced esophageal cancer which used a median cutoff value of 10 mm [8]. Although with varied tumor location, pathology, stage, statistical method and cutoff threshold, the previous studies exclusively proved the predictive value of tumor size. We adopted the medians of continuous CT variables to be cutoff values. The larger median tumor size in our study could probably be attributed to the obscurity of the cancer, misdiagnosis as other oval cavity diseases in early stage, and the lack of physical checkup for jaw lesions. The cutoff points of the preoperative tumor size as a predictor is yet to be decided to make it widely applicable.

The most appropriate cutoff of preoperative nodal size for predicting patient’s survival also remains controversial. Generally, lymph node size below 10 mm in short axis is conventionally considered non-pathologic [13]. However, other diagnostic criteria were also suggested. In a meta-analysis of head and neck cancer, size of metastatic lymph node was suggested as larger than 12 mm on CT [14]. Kawaguchi et al. adopted diameter ≥ 8 mm as a positive criterion of nodal metastasis on preoperative CT in gastric cancer patients [15, 16]. In the current study, we choose to adopt the median lymph node diameters as cutoff value instead of 10 mm in short diameter, which is a criteria for metastatic diagnosis but not for survival prediction. The univariate analysis showed that a statistically worse OS was experienced by patients with LLN ≥ 12 mm and SLN ≥ 7 mm. The multivariate analyses further proved SLN as an independent prognostic factor in patients with MC and in non-epithelial MC. Schmid et al. [5] adopted 5 mm and 10 mm cutoffs of lymph node size for patients with invasive bladder cancer. Zhang et al. [8] used a cutoff value of 10 mm for short diameter of the largest lymph node. Although different cutoff values were adopted, these studies inevitably demonstrated that preoperative nodal size on CT could predict the long-term prognosis of cancer patients. These findings suggest that the preoperative nodal status on CT is important for predicting prognosis and deciding therapeutic strategies.

The TNM staging system could be used for an estimate of prognosis in oral cancer patients [17]; however, significantly different survival rates were only observed in patients with M_1_ versus M_0_ stage in the current study, but not for different T and N stage. We did find that patients with SLN longer than 7 mm were 2.3 times more likely to have a higher T stage than those with SLN < 7 mm, while no association of LM and SLN with TNM classification was found. To be noted in the current study, all MC patients have significant OS differences based on LM and SLN, particularly prominent in non-epithelial MC patients; however, no predictive value of LM, SM, LLN, SLN and adjacent soft tissue infiltration status was found in epithelial MC patients. We have further performed analysis to compare the differences of SLN and LM between the epithelial and non-epithelial MC patients, however, no significant difference was found between the two subgroups for these two variables. Therefore, it is likely that other, as-yet-unknown factors may differently affect the survivals in both subgroups. Another explanation could be due to the small sample size, which could bias our estimates of association. Moreover, the estimated HRs could be also biased for overall MC patients and each of subgroups because of relatively small numbers of patients in each groups. Therefore, large studies are needed to confirm our findings. We did perform additional analyses restricted to epithelial cancer patients; and we found that the treatment, N stage and M stage did affect OS in this subgroup of patients. However, such a significant association was found only in 67 patients of epithelial cancers; and this finding needs to be validated in future larger studies.

Although we have confirmed the prognostic value of CT characteristics in MC patients, our study exhibits several limitations. First of all, the study design was retrospective and the data were obtained from a single institution, therefore requiring prospective and multicenter validation. Secondly, inter-observer differences in imaging assessment should be taken into account, which is usually evaluated by kappa statistic [18]. In the present study, to rule out the possible confounding from inter-observer differences, the average of three radiologists’ measurements was used for continuous variables, while the assessment of qualitative CT characteristics was conducted by consensus. The third possible limitation was the method used to configure the optimal cutoff value. As mentioned above, though with similar results, the cutoff values differed among studies. Except for the median of continuous variable as we adopted, several other approaches such as “minimum P-value approach.” [19], receiver operating characteristic curve and the Youden index [20, 21] are also statistically applicable. In addition, other parameters such as the total number of lymph nodes [8], total diameter of enlarged lymph nodes [21], metastatic nodal counts [16], and lymphadenopathy [6] have also been evaluated. Therefore, multicenter studies on larger sample size or system reviews deserve to be conducted to acquire more consistent and clinically applicable cutoffs and standards.

## Conclusions

In conclusion, preoperative CT imaging data on tumor size, lymph node size, and adjacent structure infiltration were possible predictive factors for OS of MC patients. Long diameter of the mass and short diameter of the largest cervical lymph node were independent prognostic factors in all MC, particularly in non-epithelial MC patients. The information from this study could be included when designing future preoperative monograms, and be used in clinical practice to inform patients’ prognosis.

## Abbreviations

ceCT: CT value on contrast enhanced image
CI: Confidence interval
CT: Computed tomography
HR: Hazard ratio
inCT: Increase of CT value
LLN: Long diameter of the largest cervical lymph node
LM: Long diameter of the mass
MC: Maxillary cancer
OS: Overall survival
plCT: CT value on plain image
ROI: Regions of interest
SLN: Short diameter of the largest cervical lymph node
SM: Short diameter of the mass
